# Childhood Obesity Associates Haemodynamic and Vascular Changes That Result in Increased Central Aortic Pressure with Augmented Incident and Reflected Wave Components, without Changes in Peripheral Amplification

**DOI:** 10.1155/2016/3129304

**Published:** 2016-01-11

**Authors:** Juan M. Castro, Victoria García-Espinosa, Santiago Curcio, Maite Arana, Pedro Chiesa, Gustavo Giachetto, Yanina Zócalo, Daniel Bia

**Affiliations:** ^1^Physiology Department, Faculty of Medicine, CUiiDARTE, Republic University, General Flores 2125, 11800 Montevideo, Uruguay; ^2^Pediatric Clinic “C”, Pereira-Rossell Hospital Centre, Faculty of Medicine, Republic University, Bulevar Artigas 1550, 11600 Montevideo, Uruguay; ^3^Pediatric Cardiology Service, Pereira-Rossell Hospital Centre, ASSE, Bulevar Artigas 1550, 11600 Montevideo, Uruguay

## Abstract

The aims were to determine if childhood obesity is associated with increased central aortic blood pressure (BP) and to characterize haemodynamic and vascular changes associated with BP changes in obese children and adolescents by means of analyzing changes in cardiac output (stroke volume, SV), arterial stiffness (aortic pulse wave velocity, PWV), peripheral vascular resistances (PVR), and net and relative contributions of reflected waves to the aortic pulse wave amplitude. We included 117 subjects (mean/range age: 10 (5–15) years, 49 females), who were obese (OB) or had normal weight (NW). Peripheral and central aortic BP, PWV, and pulse wave-derived parameters (augmentation index, amplitude of forward and backward components) were measured with tonometry (SphygmoCor) and oscillometry (Mobil-O-Graph). With independence of the presence of dyslipidemia, hypertension, or sedentarism, the aortic systolic and pulse BP were higher in OB than in NW subjects. The increase in central BP could not be explained by the elevation in the relative contribution of reflections to the aortic pressure wave and higher PVR or by an augmented peripheral reflection coefficient. Instead, the rise in central BP could be explained by an increase in the amplitude of both incident and reflect wave components associated to augmented SV and/or PWV.

## 1. Introduction

Blood pressure (BP) levels and wave morphology depend on a complex interplay between the heart and blood vessels. Understanding such relation is key to understanding the pathophysiology of conditions associated with increased BP [1, 2]. Schematically, mean BP (MBP) depends on cardiac output (CO) and peripheral vascular resistance (PVR), while pulse pressure (PP) is determined by the stroke volume (SV), arterial stiffness, arterial impedance, and the amplitude and sum of incident (created by ventricular contraction) and reflected components of the pressure wave [1, 2]. In turn, the regional (i.e., central versus peripheral) differences in BP levels and waveforms are mainly explained by dissimilarities in the arterial geometry, blood vessels biomechanics, and wave reflections [1, 2]. Then, different haemodynamic and vascular changes associate with an increase in BP and, hence, similar BP levels would represent different conditions in terms of ventricular afterload, target organ damage, and cardiovascular risk. For instance, increased BP in middle-aged subjects frequently associates elevated PVR and arterial stiffness, with normal or reduced SV [1]. Then, elevated systolic and diastolic BP levels are observed. On the other hand, increased arterial stiffness and the resulting increase and earlier arrival of reflected waves associate with an isolated rise in systolic BP (together with diastolic BP reduction), like that observed in the elderly [1, 2]. Additionally, “spurius or pseudo” isolated systolic hypertension observed mainly in tall and trained youth (without increased arterial stiffness) is frequently associated with an elevated central-to-peripheral PP amplification [2].

On the contrary, depending on the haemodynamic and/or vascular changes involved, the increase in peripheral BP (pBP) could take place with or without similar changes in central (cBP) BP [3–5], which raises increasing interest because of its role in cardiovascular (CV) diseases pathogenesis, predictive value for CV events, and differential response to treatment strategies [2–5].

While different haemodynamic and vascular patterns associated with changes in BP have been recognized for adults, the haemodynamic and vascular characteristics of conditions associated with increased BP in children are partially known. About this, it is recognized that obesity is frequently associated with vascular risk factors and that increased pBP levels are expected in obese children (even in those without hypertension). However, the haemodynamic and vascular profiles associated with the pBP changes and whether cBP is also modified in obese children are to be defined.

In this context, the aims of this study were (1) to determine if childhood obesity is associated with increased cBP and (2) to characterize haemodynamic and vascular changes associated with the BP variations in obese children by means of analyzing changes in cardiac output (stroke volume), arterial stiffness, PVR, and the contribution of reflected waves to the aortic pulse wave amplitude. To this end, obese and normal weight children and adolescents were studied in a noninvasive way with widely used methods (systems) to determine haemodynamic, vascular, and aortic wave derived parameters (Mobil-O-Graph, oscillometric, and SphygmoCor, applanation tonometry, systems).

## 2. Methods

All studies were approved by the Institutional Ethics Committee and written informed consent was obtained from children and adolescents guardians.

### 2.1. Study Population

Subjects included in this study were sent for evaluation in CUiiDARTE, a Uruguayan Interdisciplinary University Center that develops a program aimed at early diagnosis of arterial disease in children and adults (supported by the National Public Health Minister) [6, 7]. None of the subjects were taking medications and none had congenital, chronic, or infectious diseases at the moment of the study. All the subjects were born at term, after a healthy pregnancy.

Data were collected from 117 children and adolescents (mean age: 10 years old [range: 5 to 15 years old]; 49 females) defined as obese or with normal weight taking into account their body mass index (BMI) *z* score (*z*BMI). To obtain the *z*BMI, the calculated BMI (for each subject) was subtracted from the mean BMI in the reference population, and then the result was divided by the standard deviation for the population [8]. Individuals with a *z*BMI between −1 and 1 were assigned to the normal weight (NW) group, while those with a *z*BMI >2 were included in the obese (OB) group. Then, in order to analyze the haemodynamic and vascular characteristic associated with obesity* per se*, we defined subgroups of OB and NW without dyslipidemia, hypertension, and sedentarism (NW_wsdh_ and OB_wsdh_). Children were classified as sedentary when they performed physical activity lower than a moderate intensity physical load [8, 9]. Dyslipidemia or hypertension were considered in those children who previously were diagnosed by their referring medical doctors. Additionally, those children with pSBP and/or pDBP levels over 95th percentile of American Pediatrics Association curves reference, considering gender, age, and height, were excluded [10].

Subjects' characteristics (cardiovascular risk factors (CRF) and anthropometric data) for NW, OB, NW_wsdh_, and OB_wsdh_ are shown in Table 1.

### 2.2. Noninvasive Haemodynamic and Vascular Evaluation: Central BP, Peripheral BP, and Wave Reflection Parameters

Studies were done after 4 hours of fasting and after the medical interview and anthropometric evaluation were done. Then, individuals held a 15-minute rest in supine position in a quiet room with controlled temperature (21–23°C) before starting evaluation. Brachial BP and HR measurements were performed at fixed intervals of 8–10 minutes (HEM-433INT; Omron Healthcare Inc., IL, USA). Oscillometry (Mobil-O-Graph system) and applanation tonometry (SphygmoCor system) were used for haemodynamic and vascular evaluation.

#### 2.2.1. Oscillometric Recordings (Mobil-O-Graph System)

The Mobil-O-Graph PWA monitor system (I.E.M. GmbH, Stolberg, Germany) is a commercially available oscillometric device, validated for office (clinic) and ambulatory pBP and cBP measurement, according to the British Hypertension Society (BHS) and the European Society of Hypertension (ESH) recommendations [11]. Using the Mobil-O-Graph the aortic pulse wave velocity (PWV; an aortic stiffness index) can also be obtained (validated by referring to tonometric devices and/or invasively obtained values) [12].

The Mobil-O-Graph System's BP cuff was placed in the left arm. The device determined brachial (peripheral) systolic (SBP) and diastolic (DBP) BP levels and waveform. After that, central SBP, DBP, and PP (cPP = cSBP − cDBP) levels and BP waveforms were obtained, using a published algorithm that integrates arterial impedance and aortic haemodynamics into a mathematical model [13–15]. Form aortic waveforms and using pulse wave analysis (PWA) and wave separation analysis we obtained central aortic augmentation index (AIx), augmentation pressure (AP), forward (Pf) and backward (Pb) components of the pulse wave, and the reflection coefficient (Γ) [2, 13–15]. The merging point of incident and reflected waves (inflection point) was identified in the aortic BP waveform. AP was the difference between the maximum cSBP and the pressure at the inflection point. AIx was defined as AP divided by cPP and expressed as a percentage. Greater AIx values indicate increased wave reflection and/or earlier return of the reflected wave due to increased PWV (increased arterial stiffness) or closer reflection sites. The ARC Solver method (algorithm) allowed to calculate PWV using data derived from PWA and wave separation analysis (travel distance is estimated from body height). Finally, the cardiac output, cardiac index, and PVR were obtained [16].

Only high-quality recordings, defined as an in-device quality index >2 and acceptable curves on visual inspection, were considered. Reported values for each subject are the average of at least six consecutive records.

#### 2.2.2. Applanation Tonometry (SphygmoCor System)

Using applanation tonometry (SphygmoCor, Atcor Medical, Australia) a 10-second snapshot of the radial artery BP wave was obtained. Then, the SphygmoCor software derived the central aortic BP using a validated generalized transfer function applied to the peripheral waves [17]. Radial BP waveforms were calibrated using brachial DBP and MBP (MBP = pDBP + pPP/3). AIx and AP were derived from the aortic pressure waveform, using PWA. Only accurate waves and high-quality recordings (those with an operator index >85%, determined taking into account pulse height, and shape, height, and diastolic variations) were included.

To assess the aortic stiffness, carotid-femoral PWV (cfPWV) was calculated from sequential carotid and femoral recordings. The cfPWV was calculated as the pulse wave travel distance divided by the pulse transit time. Transit time was calculated as the difference in delays between the R wave in the electrocardiographic signal and the measured pulse waves, considering the intersecting tangents algorithm to determine the “foot of the wave” [17]. The distance between carotid and femoral recording sites used as the travel distance. A cfPWV measurement was considered valid if its standard deviation was <10%. To obtain “real” cfPWV the measured cfPWV was multiplied by 0.8 [17].

### 2.3. Statistical Analysis

Data are expressed as proportions for categorical variables and as mean value ± standard deviation for continuous variables. Student's *t*-test and chi-square test were used for comparisons of means and proportions, respectively. The criterion for statistical significance was *p* value < 0.05. All analyses were done using SPSS software version 17.0 for Windows (SPSS Inc., Chicago, IL, USA).

## 3. Results

Table 1 shows anthropometric characteristics and CRF prevalence in the different groups (NW and OB) and subgroups (NW_wsdh_ and OB_wsdh_). Note that there were no differences in sex distribution, age, or body height among subjects. Dyslipidemia and sedentarism prevalence were higher in OB than in NW (*p* < 0.05).

### 3.1. Central Blood Pressure Levels and Determinants in Obese Children and Adolescents

Table 2 shows haemodynamics and arterial parameters for NW and OB. As it was expected OB subjects showed higher pSBP and pPP levels (*p* < 0.01). In addition, cSBP and cPP levels were also higher in OB than in NW subjects. On the contrary, there were no differences in MBP and DBP between NW and OB. These findings were independent of the methodological approach considered (oscillometric system or applanation tonometry).

The amplitude of the incident (forward) aortic wave was higher in OB subjects, probably associated with increased stroke volume (*p* < 0.05) and arterial stiffness (Table 2). The net amplitude of backward (reflected) aortic wave components was higher in OB (*p* < 0.05), probably associated with the increase in the incident wave. The increase in both incident and backward wave components in OB subjects resulted in similar backward/forward ratios between NW and OB subjects (Table 2). In addition, OB and NW showed similar peripheral reflection coefficients. Then, the higher cSBP observed in OB children and adolescents could not be explained by an increase in the net (or absolute) contribution of wave reflection to central aortic pressure or by an increase in peripheral reflection coefficient. Furthermore, PVR levels were lower in OB than in NW subjects (*p* < 0.01) (Table 2).

When analyzed in relative terms, OB subjects showed a reduction in wave reflections contribution to the central BP waveform (with independence of the methodological approach considered). About this, note that reflection parameters (AP and AIx) levels were higher in NW than in OB subjects, reaching statistical significance when AIx was analyzed (Table 2).

When brachial SBP/aortic SBP ratios were analyzed, it was clear that the increase in pSBP observed in OB could not be explained by a rise in central-to-peripheral amplification.

Finally, stroke volume and cardiac output were higher in OB than in NW subjects, but when cardiac index was calculated higher values were obtained for NW subjects (Table 2).

### 3.2. Central Aortic Pressure and Determinants in Obese Subjects without Sedentarism, Dyslipidemia, and Hypertension

When children and adolescents without other classical CRF were analyzed, the results were similar to the previously reported, with independence of the methodological approach employed (Table 3). Obese children and adolescents, without sedentarism, dyslipidemia and hypertension or hypertensive BP levels during the study showed higher pSBP, pPP, cSBP, and cPP with respect to NW subjects. Additionally, with respect to NW_wsdh_, OB_wsdh_ showed higher amplitudes for both forward and backward components of the aortic pressure wave (*p* < 0.05 and *p* < 0.01, resp.) and lesser AP and AIx, despite this difference not reaching statistical significance. The higher amplitude of the incident aortic wave component could be associated with a higher stroke volume and arterial stiffness level (*p* < 0.05) (Table 3).

## 4. Discussion

Whether obesity associates with changes in central haemodynamics and/or in the arterial system properties in adults is a controversial topic. On the other hand, the information about central haemodynamics and vascular changes associated with obesity in childhood is scarce. In this context, our work main findings were as follows.

First, compared with NW subjects, OB ones had higher central (aortic) SBP and PP in addition to the expected higher peripheral BP levels. Then, the increased BP levels associated to obesity in children represent a real increase in left ventricle afterload and not only a vascular peripheral phenomenon. It is noteworthy that this finding was independent of dyslipidemia, hypertension, or sedentary habits.

Looking at our findings it could be said that the “high BP state” observed in OB children and adolescents is not an “spurious state,” due to (for instance) increased super-normal peripheral pulse pressure amplification (i.e., that reported in the isolated peripheral systolic hypertension of youth) [18]. Furthermore, giving clinical meaning to our findings, it could be said that obesity (*per se*) exposes children and adolescents to a true increase in BP and left ventricle afterload. Our findings agree with previous works in which cardiac changes consistent with response to an increased load were described in obese subjects. About this, increases in left atria [19–23] and ventricle [19, 20] dimensions were found in obese children. In addition, compared with lean controls obese children showed augmented left ventricle mass [19, 22]. Furthermore, a positive relationship between cardiac size and BMI [21, 23] has been reported in obese children. Finally, carotid intima-media thickness (which depends on BP levels) has been shown to be higher in obese children and adolescents than in those with healthy BMI [24]. Jointly analyzing our and previous findings, it could be said (at least in theory) that obesity associates haemodynamic and vascular load changes that result in/contribute to explaining the structural and functional cardiovascular changes observed in obese subjects.

Second, the increase in central and peripheral systolic pressures observed in obese children or adolescents did not associate (a) increased (relative) contribution of wave reflections to the aortic pressure, (b) higher PVR, or (c) higher peripheral reflection coefficient. In addition, the peripheral BP changes could not be explained by an increase in central-to-periphery pulse pressure amplification.

Third, the higher central and peripheral SBP and PP levels in obese children could be explained by the increase in the amplitude of both, incident and backward components of the pressure wave. The former could be associated with a rise in SV and/or aortic stiffness. In turn, the later could depend on the former increase, since, for a similar reflection coefficient, the higher the incident wave, the higher the reflected one.

The BP phenotype usually described in overweight or obese children and adolescents is that of increased peripheral systolic BP and PP, with little or no differences in diastolic BP (compared with age- and sex-matched peers with normal weight) [25–27]. Interestingly, this pBP phenotype resembles that of “isolated systolic hypertension” (ISH). About this, it is noteworthy that, in older adults, ISH is mainly associated to age-related stiffening of large central arteries, with increase in magnitude and early arrival of reflected waves [28–31]. The aortic stiffness is higher in older adults with ISH and is associated with a larger and earlier wave reflection (evident as a raised augmentation index, AIx) [31]. On the other hand, ISH in youth is frequently observed in healthy, tall, nonsmoking, and active in sports men, with slow heart rates and highly compliant central arteries. In these subjects, ISH would be secondary to an increased peripheral amplification of normal central BP.

The ISH in youth and the BP changes we found in association with childhood obesity differ. First, BP changes were observed in OB subjects whose body height, sex distribution, and heart rate were similar to those in the NW control group. On the other hand, we did not find an increased central-to-peripheral amplification or a higher relative contribution of reflected waves to central BP (levels and waveform). Instead, looking at our results it could be said that in childhood obesity the higher central and peripheral BP would be explained by increased SV (and CO) and arterial stiffness, which in turn result in augmented incident (forward) and reflected waves, despite reduced PVR and unchanged reflection coefficients. Then, the higher reflected wave could not be considered a peripheral generated phenomenon, but a central one (the higher incident wave would be the determinant of the increased wave reflections).

The higher SV observed in childhood obesity could be associated with the phenomenon known as “hyperdynamic circulation” in young individuals, which would go before the development of high BP levels [32]. Related with this, obese subjects in our study showed increased SV and CO.

Additionally, the independent influence of obesity on these differences was evident when we eliminated children with CRF. Studies showed that hypertension, sedentary, and dyslipidemia [33] are associated with increases in aortic stiffness and pB, which could affect our results.

Finally, an important aspect is that previously mentioned differences were observed using the oscillometric method as the tonometric method, which strengthens our biological findings.

## 5. Conclusions

With independence of the presence of dyslipidemia, hypertension, or sedentarism, the aortic systolic and pulse BP were higher in OB than in NW subjects.

The increase in central BP could not be explained by an increase in the relative contribution of reflections to the aortic pressure wave and higher PVR or by an augmented peripheral reflection coefficient. Instead, the rise in central BP could be explained by an increase in the amplitude of both incident and reflect wave components, associated to augmented SV and/or PWV.

Associated with this, obese children showed the highest peripheral BP levels not explained by an increased central-to-peripheral amplification.

## Figures and Tables

**Table 1:** Characteristics for normal weight (NW) and obese (OB) children and adolescents.

	NW	OB	*p* value
*N* (female %)	54 (43%)	63 (44%)	0.945
Age (years)	10.62 ± 2.93	10.34 ± 2.68	0.660
Height (m)	1.40 ± 0.15	1.44 ± 0.16	0.175
Weight (kg)	36.09 ± 11.85	58.95 ± 23.07	**0.000**
BMI percentile	61.60 ± 19.91	98.70 ± 0.83	**0.000**
*z*BMI score	0.25 ± 0.66	3.86 ± 1.59	**0.000**
Hypertension prevalence (%)	9.3	19.1	0.134
Dyslipidemia prevalence (%)	3.7	19.1	**0.010**
Sedentarism prevalence (%)	37.0	55.6	**0.045**

	NW_wsdh_	OB_wsdh_	*p* value

*N* (female %)	26 (48%)	17 (53%)	0.923
Age (years)	9.95 ± 3.15	10.2 ± 3	0.757
Height (m)	1.36 ± 0.17	1.42 ± 0.13	0.237
Weight (kg)	33.9 ± 12.5	52.1 ± 19.1	**0.000**
BMI percentile	64.2 ± 19.6	98.5 ± 1.06	**0.000**
*z*BMI score	0.26 ± 0.52	3.12 ± 0.92	**0.000**
Hypertension prevalence (%)	0	0	1.000
Dyslipidemia prevalence (%)	0	0	1.000
Sedentarism prevalence (%)	0	0	1.000

NW: normal weight; OB: obese; NW_wsdh_ and OB_wsdh_: normal weight and obese children and adolescents without sedentarism, dyslipidemia, and hypertension.

**Table 2:** Vascular parameters for normal weight (NW) and obese (OB) children and adolescents.

	NW	OB	*p* value
	MV ± SD	MV ± SD	
*Tonometry at radial artery (SphygmoCor)*			
Heart rate (bpm)	80 ± 15	77 ± 11	0.334
Peripheral SBP (mmHg)	106 ± 12	112 ± 11	**0.004**
Peripheral MBP (mmHg)	76 ± 10	77 ± 9	0.392
Peripheral DBP (mmHg)	60 ± 8	59 ± 8	0.788
Peripheral PP (mmHg)	46 ± 11	53 ± 9	**0.000**
Central SBP (mmHg)	90 ± 11	95 ± 10	**0.009**
Central DBP (mmHg)	62 ± 9	61 ± 8	0.526
Central PP (mmHg)	28 ± 9	34 ± 8	**0.000**
Augmented pressure (mmHg)	0.88 ± 3.03	−0.03 ± 4.60	0.216
Augmentation index (%)	6.47 ± 9.72	0.88 ± 10.45	**0.004**
PWV (m/s)	4.52 ± 0.70	4.62 ± 0.74	0.254
Brachial SBP/aortic SBP	1.18 ± 0.05	1.17 ± 0.05	0.361
*Oscillometry at brachial artery (Mobil-O-Graph)*			
Heart rate (bpm)	79 ± 14	77 ± 11	0.575
Peripheral SBP (mmHg)	110 ± 11	115 ± 11	**0.004**
Peripheral MBP (mmHg)	84 ± 8	86 ± 7	0.220
Peripheral DBP (mmHg)	63 ± 8	61 ± 7	0.420
Peripheral PP (mmHg)	47 ± 8	54 ± 9	**0.000**
Central SBP (mmHg)	97 ± 11	102 ± 10	**0.006**
Central DBP (mmHg)	65 ± 8	63 ± 6	0.221
Central PP (mmHg)	32 ± 8	38 ± 9	**0.000**
Augmented pressure (mmHg)	5.84 ± 3.20	5.47 ± 2.35	0.484
Augmentation index (%)	17.03 ± 8.22	14.04 ± 6.25	**0.029**
PWV (m/s)	4.45 ± 0.37	4.70 ± 0.40	**0.001**
Brachial SBP/aortic SBP	1.13 ± 0.06	1.13 ± 0.06	0.536

Reflection coefficient (%)	59.5 ± 10.3	58.7 ± 9.5	0.652
PVR (mmHg/mL·min)	1.09 ± 0.14	1.02 ± 0.15	**0.006**
Cardiac output (L/min)	4.8 ± 0.6	5.2 ± 0.8	**0.001**
Cardiac index (L/min/m^2^)	4.2 ± 0.6	3.6 ± 0.8	**0.000**
Forward pulse wave amplitude (Pf mmHg)	13 ± 4	15 ± 4	**0.003**
Backward pulse wave amplitude (Pb mmHg)	21 ± 5	26 ± 5	**0.000**
Pb/Pf ratio	0.60 ± 0.11	0.59 ± 0.10	0.811
Stroke volume (mL)	62.7 ± 14.3	69.4 ± 15.3	**0.042**

MV: mean value; SD: standard deviation; NW: normal weight; OB: obese; SBP, DBP, MBP, and PP: systolic, diastolic, mean, and pulse blood pressure; PWV: pulse wave velocity; PVR: peripheral vascular resistances.

**Table 3:** Vascular parameters for normal weight (NW_wsdh_) and obese (OB_wsdh_) children and adolescents without sedentarism, dyslipidemia, and hypertension.

	NW_wsdh_	OB_wsdh_	*p*
	MV ± SD	MV ± SD	
*Tonometry at radial artery (SphygmoCor) *			
Heart rate (bpm)	80 ± 11	79 ± 11	0.119
Peripheral SBP (mmHg)	101 ± 9	111 ± 9	**0.001**
Peripheral MBP (mmHg)	72 ± 9	79 ± 10	**0.022**
Peripheral DBP (mmHg)	58 ± 8	60 ± 8	0.445
Peripheral PP (mmHg)	43 ± 12	51 ± 7	**0.011**
Central SBP (mmHg)	86 ± 8	95 ± 9	**0.003**
Central DBP (mmHg)	59 ± 8	61 ± 8	0.431
Central PP (mmHg)	27 ± 10	34 ± 8	**0.026**
Augmented pressure (mmHg)	0.69 ± 2.99	−0.24 ± 3.38	0.351
Augmentation index (%)	3.73 ± 10.24	0.76 ± 8.70	0.331
PWV (m/s)	4.34 ± 0.69	4.68 ± 0.95	0.178
Brachial SBP/aortic SBP	1.17 ± 0.05	1.18 ± 0.06	0.860
*Oscillometry at brachial artery (Mobil-O-Graph)*			
Heart rate (bpm)	79 ± 10	74 ± 12	0.168
Peripheral SBP (mmHg)	104 ± 6	114 ± 9	**0.000**
Peripheral MBP (mmHg)	80 ± 5	86 ± 6	**0.002**
Peripheral DBP (mmHg)	60 ± 6	61 ± 5	0.550
Peripheral PP (mmHg)	44 ± 8	53 ± 5	**0.001**
Central SBP (mmHg)	92 ± 7	101 ± 11	**0.001**
Central DBP (mmHg)	62 ± 6	64 ± 5	0.386
Central PP (mmHg)	30 ± 8	37 ± 9	**0.005**
Augmented pressure (mmHg)	5.20 ± 2.87	6.07 ± 2.53	0.321
Augmentation index (%)	16.38 ± 7.46	16.06 ± 6.68	0.888
PWV (m/s)	4.25 ± 0.23	4.64 ± 0.36	**0.000**
Brachial SBP/aortic SBP	1.13 ± 0.05	1.14 ± 0.06	0.952

Reflection coefficient (%)	57.8 ± 9.5	60.0 ± 9.6	0.471
PVR (mmHg/mL·min)	1.08 ± 0.12	1.08 ± 0.15	0.966
Cardiac output (L/min)	4.6 ± 0.5	4.9 ± 0.7	0.084
Cardiac index (L/min/m^2^)	4.2 ± 0.6	3.6 ± 0.9	**0.018**
Forward pulse wave amplitude (Pf mmHg)	12 ± 4	15 ± 4	**0.014**
Backward pulse wave amplitude (Pb mmHg)	20 ± 4	25 ± 5	**0.003**
Pb/Pf ratio	0.58 ± 0.10	0.60 ± 0.10	0.608
Stroke volume (mL)	60.1 ± 12.9	69.1 ± 13.9	**0.043**

MV: mean value; SD: standard deviation; NW_wsdh_ and OB_wsdh_: normal weight and obese children and adolescents without sedentarism, dyslipidemia, and hypertension; SBP, DBP, MBP, and PP: systolic, diastolic, mean, and pulse blood pressure; PWV: pulse wave velocity; PVR: peripheral vascular resistances.
